# Symbiosis, Parasitism and Bilingual Cognitive Control: A Neuroemergentist Perspective

**DOI:** 10.3389/fpsyg.2018.02171

**Published:** 2018-11-19

**Authors:** Arturo E. Hernandez, Hannah L. Claussenius-Kalman, Juliana Ronderos, Kelly A. Vaughn

**Affiliations:** Department of Psychology, University of Houston, Houston, TX, United States

**Keywords:** bilingualism, cognitive control, development, computational models, language acquisition

## Abstract

Interest in the intersection between bilingualism and cognitive control and accessibility to neuroimaging methods has resulted in numerous studies with a variety of interpretations of the bilingual cognitive advantage. Neurocomputational Emergentism (or Neuroemergentism for short) is a new framework for understanding this relationship between bilingualism and cognitive control. This framework considers Emergence, in which two small elements are recombined in an interactive manner, yielding a non-linear effect. Added to this is the notion that Emergence can be captured in neural systems using computationally inspired models. This review poses that bilingualism and cognitive control, as examined through the Neuroemergentist framework, are interwoven through development and involve the non-linear growth of cognitive processing encompassing brain areas that combine and recombine, in symbiotic and parasitic ways, in order to handle more complex types of processing. The models that have sought to explain the neural substrates of bilingual cognitive differences will be discussed with a reinterpretation of the entire bilingual cognitive advantage within a Neuroemergentist framework incorporating its neural bases. It will conclude by discussing how this new Neuroemergentist approach alters our view of the effects of language experience on cognitive control. Avenues to move beyond the simple notion of a bilingual advantage or lack thereof will be proposed.

## Introduction

Bilinguals vary tremendously in the ways in which they learn two languages. Some learn a second language in childhood, adolescence, or adulthood while others may learn two languages in infancy. They may also vary in the ways in which these two languages are used with some involving formal schooling and others being mostly spoken languages. This naturally brings up the question of how a bilingual manages these two languages. To account for this, the notion of a language switch was proposed, adapting the notion of a switch first proposed by Penfield (1965) (Penfield and Roberts, 1959). The notion of a language switch was the inspiration for a greater number of studies across at least four decades (Hernandez, 2013). More recently, this debate has taken on a more modern nomenclature by considering the nature of cognitive control and its role in managing two languages.

Despite the long history of linking cognitive control to bilingualism, recent discussion has become contentious (Hilchey and Klein, 2011; Abutalebi et al., 2012; Hernández et al., 2013; Paap and Greenberg, 2013; Anton et al., 2014; Paap et al., 2014; de Bruin et al., 2015; Duñabeitia and Carreiras, 2015). Seminal studies in several labs have found that being exposed to two languages is associated with better performance on non-verbal cognitive control (Bialystok, 2007). Moving beyond the notion of language switching and its neural bases, work in the neuroimaging literature supports the view that bilingualism has the potential to strengthen frontal-striatal pathways due to the constant use of more than one language (Abutalebi et al., 2007, 2012; Green and Abutalebi, 2013; Stocco and Prat, 2014; Stocco et al., 2014) leading to advantages in cognitive control (Bialystok et al., 2004, 2007; Engel de Abreu et al., 2012; Bradley et al., 2013; Buchweitz and Prat, 2013; Marian et al., 2014). However, there is still considerable debate about whether the learning and use of two languages leads to a non-verbal cognitive control advantage in bilinguals (Hilchey and Klein, 2011; Abutalebi et al., 2012; Hernández et al., 2013; Paap and Greenberg, 2013; Anton et al., 2014; Paap et al., 2014; de Bruin et al., 2015; Duñabeitia and Carreiras, 2015). This has led many in the field to propose that we stop investigating the role that bilingualism may play in non-verbal cognitive control.

Despite the calls for completely abandoning the notion of a “bilingual advantage,” there is ample evidence that cognitive control and bilingualism are intimately linked. For example, work with older adults has found a posterior-to-anterior shift in normal aging (Davis et al., 2008; Dennis et al., 2008). The posterior-to-anterior shift is consistent with the view that older adults engage brain systems involved in cognitive control to a greater extent than young adults when performing the same task. Recent work in the bilingual literature finds the opposite pattern showing an anterior-to-posterior shift in bilinguals relative to monolinguals (Grant et al., 2014). The finding that bilingualism leads to the opposite, anterior-to-posterior (and subcortical) effect, has led researchers to consider whether bilingualism may serve as a cognitive protective factor (Grundy et al., 2017). The notion of a simple bilingual advantage has also led researchers to consider the variety of experiences including the age of acquisition, amount of language use, amount of switching, individual differences in language ability, and individual differences in flexibility and working memory that might influence the control mechanisms that are used in various non-verbal tasks (Green and Abutalebi, 2016; Yang et al., 2016; Birdsong, 2018). With all these different influences collectively pushing and pulling on the development of a bilingual’s two languages, a number of researchers have proposed that bilingualism has the characteristics of a non-linear dynamical system (Hernandez et al., 2005; Hernandez and Li, 2007; De Bot, 2008; Hernandez, 2013; Li et al., 2014).

In this piece, we build on this work using the notion of Neurocomputational Emergentism (Neuroemergentism) to provide a framework within which we can better understand the neural bases of cognitive control in bilinguals within a developmental framework. This approach seeks to consider Emergence, in which two small elements are recombined in an interactive manner, yielding a non-linear effect. Added to this will be the notion that this Emergence can be captured in neural systems using computationally inspired models. While Neuroemergentism has been applied more directly to the content of language (i.e., grammar, semantics, etc.,) it is likely that these diverging and converging influences affect cognitive control as well. We will begin by reviewing the field of Emergentism before proceeding to consider the nature of this within a computational neuroscience view that takes into account the developmental processes associated with the learning of two languages.

## Emergentism

The notion of emergent function has its roots in the work of Mill et al. (1974) who used examples from chemistry to argue that combining two simple things can lead to a much more complex form. For example, combining hydrogen and oxygen results in a new compound, water, due to the transformation of two gaseous elements into a liquid. The nature of dynamical changes seen in emergent forms was also considered by Bates et al. (1979); Bates (1999), who, inspired by the work of Thompson (1917) on the mathematics of physical transformation in the animal kingdom, proposed that language itself involved building “a new machine out of old parts.” The fact that old things may recombine into a newer whole can also be seen in other analogies used by Bates (1999). For example, one analogy included the giraffe’s neck, an adaptation to having to eat leaves high up in a tree that led to a number of cascading changes in the cardiovascular system and even the size and distribution of the hind and forelegs in order to conserve its balance. Thus, a small change could lead to changes across multiple different anatomical systems. This Emergentist view was further developed in the Competition Model, which sought to explain how grammar emerged from coalitions and rivalries between cues at one level that were then recombined to create a complex grammar that signaled who did what to whom at a much higher level (Bates and MacWhinney, 1981).

Work within the framework of the competition model considered the case of bilingualism. Bates and MacWhinney (1981) argued that a second language could potentially interact in a number of ways, including the bidirectional influences of first and second language grammatical processing termed forward and backward transfer, respectively. Amalgamation in which the grammars of both languages are fused together and differentiation in which each grammar is kept apart were also viable possibilities. Results from a series of studies on bilingual grammatical processing provided support for both forms of transfer as well as for amalgamation and differentiation.

In 2005, Hernandez et al. (2005) built on the Competition Model to propose an Emergentist framework that describes the acquisition of two languages. In that paper, the authors argued that through competitive interplay, children would learn to map form to meaning in each language and adjust it according the situation. For example, a child might learn to use a word form “taza” or “cup” depending on the language of a speaker. In a similar fashion, a bilingual model of vocabulary learning based on a model of vocabulary acquisition in a single language revealed that exposure to English and Mandarin at an early stage leads to two independent representations (Zhao and Li, 2006). When the network was exposed to a second language (either Mandarin or English) at a later point in time, the second language became parasitic on the first. This competitive process is also reminiscent of Hernandez’s (2013) view that a bilingual’s two language are like two species in a single ecosystem that can compete for or share resources depending on the situation.

To date, the Emergentist view has mostly focused on the outcome of language processing, including the learning of new vocabulary as well as grammar, using a cognitive framework. However, this view can offer a framework with which to try and make sense of a newer question: whether bilinguals have some cognitive advantage relative to monolinguals. Although Emergentism does not make particular predictions about cognitive control, it can be extended to consider how the competitive process plays out across time resulting in the use of control to dampen it down. Furthermore, this framework also suggests that the nature of the competitive interplay across languages will differ depending on the age of acquisition of a second language.

Hernandez and Li (2007) were the first to propose a neurocomputational approach for the dynamic nature of change across time to explain age of acquisition effects across multiple domains. They suggested that learning earlier and later in life would rely on different neural and cognitive systems. Whereas learning early in life relied to a greater degree on neural systems involved in sensorimotor processing, later learning would rely on association areas which were used to bind sensory information or to combine it with motor processing. Computationally, early AoA effects led to distinct representations for each language whereas later learning resulted in a parasitic relationship in which the second language was built around the first (Zhao and Li, 2006).

One limitation of Hernandez and Li’s (2007) Sensorimotor Hypothesis is that it might be mistakenly interpreted as suggesting that early learning only has an effect early in life. To overcome this static interpretation of age of acquisition, a newer framework, Neuroemergentism has been introduced. This framework is based on the idea that development involves the non-linear growth in brain areas that combine and recombine information in order to handle more complex types of processing. As such effects early in life can carry repercussions later in life. In the case of bilingualism, it is the case that early experiences can leave the neural substrate open to new experiences later in life. We will return to this point later after discussing the types of new questions that existing frameworks of cognitive control in bilingualism are opening up for researchers.

In the following sections, a summary and discussion of the bilingual advantage will be presented. A discussion of the models that have sought to elucidate the neural substrates of bilingual cognitive differences will also be discussed. The piece will end with a reinterpretation of the entire bilingual cognitive advantage within a Neuroemergentist framework incorporating its neural bases. It will conclude by discussing how this new Neuroemergentist approach alters our view of the effects of language experience on cognitive control. Avenues to move beyond the simple notion of a bilingual advantage or lack thereof will be proposed.

## The Bilingual Cognitive Advantage: Findings and Models

The notion of a bilingual cognitive advantage began most recently in the mid-2000’s when a number of research studies with different age groups found that bilinguals outperformed monolinguals across a variety of cognitive control tasks (Bialystok, 2006; Bialystok et al., 2004, 2007). Based on these findings, researchers concluded that the bilingual experience leads to an improvement in the ability to use executive control. In recent years, this claim has met considerable skepticism due to evidence both at the behavioral and neural levels (Morton and Harper, 2007; Paap, 2012; Kousaie et al., 2014; de Bruin et al., 2015). Given the variability in findings across studies, one is left wondering whether the advantage exists or not (for further discussion see Bialystok et al., 2015).

Research investigating the bilingual advantage is based to a greater extent on behavior; the brain mechanisms underlying potential differences in the use of cognitive control in bilinguals relative to monolinguals have not received nearly as much attention. Whether language experience results in behavioral differences or not, it is entirely possible that learning two languages results in differences at the neural level (Vaughn et al., 2015). Furthermore, researchers have begun to offer theoretical models that account for the effects that bilingual experience has on the cognitive control system, including the Bilingual Adaptation Model, the Adaptive Control Hypothesis (ACH) and the “Brain Training” model. Examining these three models in terms of their focus on cognitive changes or neural changes associated with bilingualism: the Bilingual Adaptation Model is based mostly on cognitive changes, the ACH is based on both cognitive and neural changes, and the Brain Training Model is based mostly on neural changes.

The Bilingual Adaptation Model, proposed by Bialystok (2017) suggests that being raised in a bilingual environment enables the development of a more flexible attention system associated with frontal brain regions. This model focuses on behavioral findings, such as gaze direction in infants and task performance in children, and suggests that, in general, the neural adaptations associated with executive attention in bilinguals overlap with language processing and selection. The Bilingual Adaptation Model includes aspects of Engle (2002) model of working memory capacity, as well as Posner and Petersen’s (1990) sustained, selective, and executive attention networks.

The ACH, posited by Green and Abutalebi (2013), focuses on different bilingual environments and neurological and cognitive adaptations related to those environments. For example, the executive functions needed for interacting in a single language context are different from those needed to interact in a dual-language context and those needed to interact in a dense code-switching context. Three potential neural mechanisms may account for the improvements in executive function as a result of the language contexts: “through a change in structural resources or capacity (e.g., gray matter density), through a change in regional efficiency (e.g., through tuning neuronal populations or changing the responsiveness of neuronal populations) or through a change in the connectivity of the network (e.g., white matter connectivity)” (p. 517). The specific networks altered by bilingualism depend on the cognitive functions being altered, which depend on the language context. Finally, the ACH highlights the importance of the frontal-striatal tract in learning two languages, and also considers other areas including the SMA, ACC, and inferior parietal lobule.

The importance of the striatum is the topic of the “brain-training” model (Stocco and Prat, 2014). Stocco et al. (2014) suggest that the basal ganglia is particularly well-equipped to handle bilingual language switching linking this phenomenon to earlier work with the conditional routing model (Stocco et al., 2012). During the learning process or in situations where automatized cognitive routines cannot accomplish a task (for example, in task-switching), the basal ganglia can amplify signals from a selected source region to enhance the likelihood that this signal will influence behavior despite weaker cortico–cortical network connections. This model also fits with more recent work that has begun to consider the role of dopamine and dopamine-related genes in cognitive flexibility and stability.

## Development: the Missing Piece

Despite calls for researchers to take into account the variability in the bilingual experience, there has been a paucity of work on the developmental mechanism or mechanisms that might contribute to cognitive control. Hernandez and Li (2007), although not focused on cognitive control *per se*, provide a foundation for considering this topic. In their review of the literature, they noted that human development is characterized by successive waves of change that begin in areas that are devoted to sensorimotor processing, proceed to multisensory integration areas and end with the development of the prefrontal cortex which binds the senses and motor responses, paving the way for complex forms of cognition. One important prediction is that early learning involves greater potential for structuring the building blocks of a much simpler system which carries with it effects at a higher level. For example, age of acquisition effects, which have been linked to brain areas involved in phonological and prosodic processing, could then “spill over” to the processing of grammar. Age of acquisition effects in grammatical processing could be the outcome of the warping of sound space to particular combinations of both speech and prosodic patterns that contain grammatical information. Thus, the loss of plasticity in areas involved in sensorimotor processing can have pervasive effects beyond that level into other higher-level domains. As neural connections are solidified, plasticity is lost (for a more extensive discussion see Hernandez and Li, 2007).

Interestingly, this developmental view of sensorimotor integration during early ages also sheds light on the possible effect of early and later experience with a second language on cognitive control. Here we will offer two examples that elucidate the ways in which development can contribute to our understanding of the relationship between cognitive control and the learning of two languages. We will base our discussion around the notion of an anterior to posterior/subcortical shift and its roots in a developmental model of bilingualism.

## Symbiosis, Parasitism and Cognitive Control

Simultaneous bilinguals provide one of the most interesting test cases for theories of language development. Learners of two languages in infancy reveal utterances that are well organized and toddlers are able to adapt to the language output of an adult speaker (Genesee et al., 2008). At the same time, they can code-switch and produce utterances with words from both languages. Catch them early enough and infants exposed to two languages will even babble in each language, producing utterances that sound like one or the other language (Andruski et al., 2013). The brain must adapt to these two systems from the beginning of life.

How might these early language effects influence cognitive control? One mechanism proposed by Bialystok (2017) is that of executive attention, which bilingual infants use to focus on the mouths of speakers. Another mechanism would be at the speech categorization level. For example, Krizman and Marian (2015) suggest that the auditory system is intimately tied in with the executive control system via the basal ganglia up into cortical areas involved in cognitive control including the anterior cingulate cortex (ACC) and the dorsolateral prefrontal cortex (DLPFC). There are also feedback loops from the ACC back down toward the basal ganglia. If one considers an individual who learned two languages from a young age, it is likely that adaptations at the level of the basal ganglia would have happened due to the exposure to two distinct phonological and prosodic patterns for each language. Thus, simultaneous bilingualism would lead to a distinct set of neural signatures in subcortical structures and in the basal ganglia in particular. Early childhood bilingualism might involve attentional areas needed to distinguish speakers of each language and to map space onto language use. Later second language acquisition would involve the prefrontal cortex to a greater extent, in order to overcome the preponderant responses of the first language. In this view, the pre-frontal cortex would become involved in order to overcome an entrenched second language that might be parasitic on the first language. This competitive and cooperative view of language development at various ages takes us back to the notion of development as being characterized by cascading interactions at various neural levels, the hallmark of a Neuroemergentist approach.

One final piece of this puzzle is to consider neurocomputational models and how these might fit in with a Neuroemergentist approach. Only one model of bilingual cognitive control, Stocco’s “brain training” model has a neurocomputational implementation (Stocco et al., 2014). One interesting aspect is that Stocco’s model is not framed around cognitive control *per se* but rather around reinforcement learning. In this vein, the basal ganglia are recruited during the learning process or in when automatized cognitive routines are unable to successfully complete a task (for example, in task-switching). In these cases, the basal ganglia can amplify signals from a selected source region to enhance the likelihood that this signal will influence behavior despite weaker cortico–cortical network connections.

That suggests that language history plays a role in learning was conducted by Bradley et al. (2013). In that study, a group of Spanish-English bilinguals and monolinguals were asked to learn a set of “new” German words via translation. After reaching 90% correct, they were placed in the scanner and asked to make a living/non-living judgment. The results revealed better performance in the bilinguals in that they had lower reaction times relative to monolinguals. In addition, bilinguals showed increased activity in the putamen whereas monolinguals showed relatively greater activity in the caudate nucleus and cortical cognitive control areas. All of our bilingual subjects had learned English relatively early in life. Thus, these results are consistent with the view that an early age of second language acquisition leads to neural adaptations when learning a new task. Together with Stocco’s neurocomputational model they would suggest that age of acquisition is not only affecting the content of cognition, it may be also affecting the way in which the brain handles new learning. Future studies could use a combination of brain and computational science to further examine this question.

## A Final Note on Non-Linear Dynamics

So far, our Neuroemergentist view, which has focused on the sensorimotor aspect of early learning which differs from later learning, suggests that signatures would include a dynamic interaction between an individual and his or her environment. The nature of this dynamic Neuroemergentist view can be seen in the surprising result from a study on biomarkers and behavioral responses in a group of middle-age monolingual and bilingual participants in which early bilingualism was associated with better performance on tasks of executive function (Estanga et al., 2017). The early bilingual group was also found to have a lower presence of t-tau levels in their cerebrospinal fluid (CSF). The presence of a biomarker in the CSF is an unlikely place to search for an effect of early bilingualism. However, this biomarker is one that has received considerable attention in the literature. One area that is subject to tau-pathology (Grudzien et al., 2007), the locus coeruleus, has been posited to play a role in cognitive reserve via noradrenergic stimulation (Mather and Harley, 2016). Furthermore, the locus coeruleus, tyrosine-hydroxylase-expressing (THþ) neurons have been found to mediate post-encoding memory enhancement, possibly through the release of dopamine in the hippocampus (Takeuchi et al., 2016), another structure of crucial importance for Alzheimer’s disease.

The fact that changes in the CSF are associated with language learning history opens up two questions. The first revolves around age of acquisition, a key topic that was discussed at length in previous Neuroemergentist pieces (Hernandez et al., 2005; Hernandez, 2013; Hernandez and Li, 2007; Hernandez et al., In press). If early acquisition of two languages involves earlier-developing neural systems, then one is left wondering whether cognitive reserve in older adults is due to the continued use of two languages across a long period of time or due to the age at which second language exposure began. The fact that this effect differed between two groups of middle-age individuals with significant length of exposure and use of two languages is consistent with the view that that age of acquisition and not use of both languages may be leading to greater cognitive reserve. This fits in with the view that learning early in life is much more embodied, a view that has been found in a number of domains both between, within, and outside of language (Hernandez and Li, 2007; Hernandez et al., 2011).

Recently, we have proposed the term Neuroemergentism to suggest that these non-linear interactions occur at the level of the brain. To be clear, other researchers have suggested similar approaches. This Neuroemergentist approach does share features with Neuroconstructivism (Karmiloff-Smith, 2009, 2015) and the Interactive Specialization (Johnson, 2011) approaches that have been proposed by Karmiloff-Smith, Johnson and colleagues. However, Neuroemergentism emphasizes the appearance of effects from the combination of much smaller systems. This fits with the examples given earlier. Namely, that low level interactions in the auditory system of early bilinguals as well as possible effects of arousal, attention and/or memory handled by the locus coeruleus in concert with a wide range of other brain areas, would lead to the presence of a pervasive effect that lasted into middle age and beyond. The question remains of what late bilingualism brings to the table. Here, our contention is that these effects are more likely to appear in cortical systems and work their way down to subcortical systems, leading to significant rewiring (Li et al., 2014).

One question that remains with regard to Neuroemergentism is what tangible alternatives it might offer compared to other frameworks. First, because of its emphasis on the non-linear dynamics of change, it takes into account developmental variables that have not been traditionally considered in the ACH of Abutalebi and Green as well as the Executive Attention proposal of Bialystok. These developmental effects are likely the product of varying forms of bilingual experience that could be studied as well (Antoniou, 2018). Both of these generally do not consider how development might contribute to cognitive control. Second, our framework seeks to look at how cognitive control is built up across time. This non-linear process has potential weakness in that it could be seen as explaining any particular non-linearity that might appear using the veil of Emergentism an apostierori explanation. To overcome this limitation, Neurocomputational Emergentism, actually seeks to find specific causes for emergent behavior due to neural reorganization. One specific example would involve the use of the “brain training” model of the basal ganglia as proposed by Stocco and colleagues. It could be adapted to handle linguistic input during development. Additional experiments with children could then be used to look at the effects of second or dual-language learning on cognitive control. This would in turn generate new predictions. This cycle of experimental observation of emergent behavior, modeling of that behavior and new predictions would eventually allow a more complete view of what the causes of any particular advantage in cognitive control might be. Although this framework is in an early phase of development at this time, the issues discussed in this piece point to the benefits of a Neuroemergentist approach to this question.

However, rather than seeing bilingualism as a unique contributor, within this Neuroemergentist view we would argue that it serves as a window within which researchers can observe the interconnectedness of neural systems that are thought to be dissociated. In short, experience with two languages does more than alter the neural substrate responsible for language. Rather, the effects of language experiences are likely pervasive, and not easily reduced to a simple reaction time advantage or a single pattern of brain activity. In addition, the case of adoptees goes beyond a simple bilingual/monolingual dichotomy. For example, neural traces have been observed in adults even when there is no conscious knowledge of a language that was discontinued very early in childhood (Pierce et al., 2015). Future endeavors should continue to use traditional methods in behavioral and brain science along with computational models that can handle the type of non-linear interactions seen in development. The remaining question, thus, should not center on whether language experience at different points in life affects or does not affect cognition. The question is how it does. A question that with the help of dynamic developmental frameworks such as Neuroemergentism is likely to keep researchers occupied for years to come.

## Author Contributions

AH contributed to the introduction and conclusion as well as with the overall organization of the entire manuscript. HC-K contributed to discussion of bilingualism and its relationship with attention and manuscript formatting. JR contributed to the discussion of Neuroemergentism in the context of bilingual language development and manuscript formatting. KV contributed to the overview of the bilingual cognitive advantage and assisted with formatting the manuscript according to Frontiers standards.

## Conflict of Interest Statement

The authors declare that the research was conducted in the absence of any commercial or financial relationships that could be construed as a potential conflict of interest.
